# Mobile Applications in Clinical and Perioperative Care for Anesthesia: Narrative Review

**DOI:** 10.2196/25115

**Published:** 2021-09-17

**Authors:** Sabrina Pan, Lisa Qia Rong

**Affiliations:** 1 Weill Cornell Medicine New York, NY United States; 2 Department of Anesthesiology Weill Cornell Medicine - New York-Presbyterian Hospital New York, NY United States

**Keywords:** mobile applications, mHealth, perioperative medicine, anesthesia

## Abstract

**Background:**

The increasing use of smartphones by providers and patients alike demonstrates that digital health utilizing mobile applications has the potential to transform perioperative care and education in anesthesia.

**Objective:**

This literature review describes the current scope of the use of mobile applications in anesthesiology.

**Methods:**

Literature was searched using PubMed, Scopus, and clinicaltrials.gov for articles published from January 1, 2010, through April 1, 2020. Only English language studies were included. Articles were included if they examined the use of a mobile health application in the setting of anesthesia or the perioperative (immediate preoperative, intraoperative, and postoperative) period. Studies were excluded if they explored video interventions or did not examine the feasibility or efficacy of the mobile app.

**Results:**

We included 29 articles, and three areas of clinical functionality were identified: patient-centered care (preoperative, intraoperative, and postoperative), systems-based improvement, and medical education. Several studies demonstrate the feasibility and reliability of mobile apps in these areas, but many are only tested for efficacy in simulated environments or with small patient samples

**Conclusions:**

Mobile health applications show promise in improving communication between anesthesiologists, improving workflow efficiency, enhancing medical education, and reducing hospital costs. However, there is a need for validation and improvement before full implementation by the provider, patients, and hospital systems. Future studies are needed to demonstrate meaningful health outcomes to create guidelines and recommendations specific to the application of mobile technology to health care.

## Introduction

Mobile health (mHealth)––digital health technologies using mobile phones, tablets, and wearables to improve health outcomes––have the potential to rapidly change the face of medicine, health care, and medical education. By 2019, 81% of Americans owned a smartphone, a significant increase from 35% in 2011 [1]. Additionally, there has been an increased level of interest in using mobile apps to monitor health in the US population, with 45,028 mHealth related applications available for download in Apple’s App Store in 2019, up from 28,343 in 2015 [2]. New advances in mobile apps provide opportunities for innovative patient care, real-time data delivery, and patient-provider engagement in anesthesiology.

Previous reviews in anesthesia have discussed commercially available digital health apps provided in the Apple App Store or Google Play market [3]. This review, however, describes recent clinically tested digital health apps found in scientific literature and how these tools may be integrated into anesthesiologists’ clinical practice.

This review aimed to describe the current scope of mobile application use within anesthesiology. We classified apps found in recent scientific literature into areas of clinical functionality that were deduced from the analysis of the included studies. We also described the limitations of mobile application studies and suggested future directions.

## Methods

### Overview

A review was conducted to answer the following research question: What mHealth applications in anesthesiology have been described in the published literature, and what are their utility and future potential in clinical practice?

### Database Search

A complete literature search was conducted with the librarian's assistance using PubMed, Scopus, and clinicaltrials.gov for articles published from January 1, 2010, through April 1, 2020. The search terms were “mobile applications” OR “mobile health” OR “digital health” OR “mobile app” OR “iOS app” OR “mHealth” OR “smartphone” AND “anesthesiology” OR “anesthesia” OR “anesthetic” OR “anesthesiology” OR “anesthesia” OR “anesthetic. The search included titles, abstracts, and keywords for all databases.

### Article Selection

Title, abstract, and full text of studies were reviewed by the first author (SP), and the senior author (LR) resolved any ambiguities. Only English language studies were included. Articles were included if they examined the use of an mHealth application in the setting of anesthesia or perioperative evaluation. Studies were excluded if they explored video interventions or did not examine the feasibility or utility of the mobile app.

### Data Collection and Summary

Each article was assessed by design, population characteristics, methods, area of clinical functionality, and outcomes. A qualitative analysis was then conducted to summarize the depth and breadth of mHealth apps that have been utilized within anesthesiology. Major clinical functionalities were deduced from this qualitative analysis, and each study was then classified into one of these categories. We then described the limitations of the data from these studies and provided a basis for the future direction of mHealth in the field.

## Results

Our search returned 80 studies on PubMed, 141 on Scopus, and 5 studies on clinicaltrials.gov, yielding a total of 226 studies, of which 49 were duplicate studies. The titles and abstracts of the remaining 177 articles were screened. We identified 66 that meet the criteria, and their full text was reviewed, after which 29 articles were selected for a qualitative review.

Within the literature, we identified three areas of clinical functionality: patient-centered care, systems-based improvement, and medical education. Patient-centered care was further subdivided into preoperative, intraoperative, and postoperative monitoring. Certain mobile applications within systems-based improvement were also subcategorized into medication safety or adverse event reporting or guideline compliance. An overview of each study's characteristics, methods, and outcomes can be found in Table 1.

**Table 1:** Characteristics and outcomes of included studies.

Study	Design	Sample, N	Sample characteristics	Category	Methods	Results
Cumino et al 2017 [4]	RCT^a^	84	Pediatric (4-8 years) undergoing elective surgeries	Patient-centered care: Preoperative	Four groups: control (verbal anesthesia), informed (parent given an information leaflet about the anesthetic procedure), smartphone (with a child in holding area), and combined (smartphone or informed). Primary endpoint: m-YPAS^b^ in holding area and OR^c^	The OR anxiety in the control group was higher (76.2%; *P=*.001) than in the other groups: informed group (38.1%), smartphone group (23.3%), and combined (19.0%).
Lee et al 2013 [5]	RCT	120	Pediatric (1-10 years) surgical patients	Patient-centered care: Preoperative	Midazolam (M group), smartphone app (S), or midazolam & smartphone (SM). Primary outcome: m-YPAS before and after the intervention.	Group S score was significantly lower than group M (*P=*.01, group SM was significantly lower than group M (*P<*.01), and group SM was significantly lower than group S (*P<*.01).
Rubin et al 2019 [6]	Prospective	86	Adult surgical patients	Patient-centered care: Preoperative	An app was designed to administer the Duke Activity Status Index and the 6-minute walk test. Linear regression was performed to estimate the distance walked during the 6MWT from the number of steps measured by the app.	Steps measured by app and research-grade pedometer demonstrated intraclass correlation of 0.87 (0.79-0.92; *P*<.001). Overall model fit was r^2^=.72 for the distance estimation algorithm.
Barrachina et al 2017 [7]	Cross-sectional	20	Intensive care unit patients	Patient-centered care: Intraoperative	Analysis of snapshots from patient monitor and photos using Capstesia app were assessed for concordance of PPV^d^, CO^e^, and dP/dt^f^	Intraclass correlation coefficient or PPV, CO and max dP/dt were 0.991 (95% CI 0.98-0.99), 0.966 (0.96-0.97) and 0.96 (0.95-0.97), respectively.
Carvalho et al 2019 [8]	Prospective	22	Patients with surgery requiring NMB^g^	Patient-centered care: Intraoperative	NMB grade assessed intraoperative care with TOF^h^ ratios obtained by a Stimpod accelerometer versus the new app	For 142 time points, there was no significant difference between the two methods (*P=*.78). However, insufficient data that the app can diagnose a TOF higher than 0.
Desebbe et al 2016 [9]	Prospective	2100	N/A^i^	Patient-centered care: Intraoperative	The simulator is used to display arterial waveforms on a computer screen. Data was obtained with different sweep speeds (6 and 12 mm/s) and randomly generated PPV values, pulse pressures, and vitals. Each metric was recorded 5 times at an arterial height scale X1 and 5 times at an arterial height scale X3. Primary outcome: Reproducibility of PPV_app_^j^ and PPV_man_^k^.	The precision error of PPV_app_ and PPV_man_ was 10% (7%-14%) and 6% (3%-10%), respectively. PPV_app_ shows acceptable accuracy with PPV_man_ when at least three pictures are taken to average PPV_app_ at scale X1 (upper limit of the 95% CI of the measurement error<12%).
Joosten et al 2019 [10]	Cross-sectional	40	Abdominal surgery patients	Patient-centered care: Intraoperative	PPV_CAP_^l^ compared with SVV_PC_^m^ at postinduction, preincision, postincision, the end of surgery, and during every hypotensive episode. PPV and SVV are classified into no fluids, gray zone, and fluid administration. Primary outcome: the overall agreement between PPV and SVV and agreement in the fluid administration category	549 pairs of PPV-SVV data were obtained. The overall agreement of PPV_CAP_ with SVV_PC_ was 79% ( κ=0.55), demonstrating moderate agreement with only 1% of all measurements resulting in opposite clinical decisions regarding fluid administration.
Joosten et al 2019 [11]	Prospective	57	Elective coronary artery bypass graft patients	Patient-centered care: Intraoperative	The ability of PPV_CAP_ or PPV_PC_^n^ to predict fluid responsiveness after infusion of 5 ml/kg of colloid. Primary outcome: overall agreement of PPV_CAP_ and PPV_PC_ as well as CO_CAP_^o^ and CO from CO_TD_^p^	No difference in the ability of PPV_CAP_ and PPV_PC_ to predict fluid responsiveness (AUROC^q^ 0.74, 95% CI 0.60-0.84 vs AUROC 0.68, 95% CI 0.54-0.80; *P =*.30). CO_CAP_ did not correlate well with CO_TD_.
Chiu et al 2019 [12]	RCT	156	Procedural patients, age 19-75 years,	Patient-centered care: Preoperative	Completed VAS-100^r^ and NRS-11^s^ for pain using paper versus smartphone VAS-100 and NRS-11. Primary outcome: correlation at various time points	Panda VAS-100 and original tool correlated strongly at emergence (r =.93) and upon discharge (r =.94); Panda NRS-11 correlated strongly with the original at emergence (r =.93) and upon discharge (r =.96)
Dahlberg et al 2017 [13]	RCT	1027	>17 y/o undergoing day surgery	Patient-centered care: Postoperative	Patients answered SwQoR^t^ daily for 14 days: smartphone app versus standard care (control). Primary outcome: cost-effectiveness.	Net savings of €4.77 (approximately US $5.65) per patient w/ intervention. No difference in SwQoR between the two groups.
Dahlberg et al 2019 [14]	RCT	494	>17 years undergoing day surgery	Patient-centered care: Postoperative	Patients randomized to RAPP^u^ daily for 14 days via app versus standard care (no follow-up).	62% of contacts made by patients were on postoperative days 1 to 7 and 38% on postoperative days 8 to 14. Demonstrated follow-up should be more long-term.
Highland et al 2019 [15]	RCT	50	Military surgery patients	Patient-centered care: Postoperative	Mobile app (mCare) group vs control telephone group to complete Defense and Veterans Pain Rating Scale, nerve block questions, satisfaction surveys, and system usability survey after surgery.	No difference in initial response rates between app and control. On day 8, 64% of mCare group completed follow-up versus 48% of the control group (*P=*.29).
Jaensson et al 2017 [16]	RCT	997	Adult day surgery patients	Patient-centered care: Postoperative	SwQoR daily for 14 days via app intervention (RAPP) or standard care (control). Primary outcome: SwQoR on postoperative days 7 and 14	Global SwQoR score was significantly lower (better recovery) in the RAPP group compared to the control group on day 7 (28.23 vs 34.87; *P<*.001) and day 14 (20.12 vs 21.90; *P=*.002).
Ke et al 2019 [17]	Qualitative study	15	Obstetric anesthesiologists (9) and cesarean section patients (15)	Patient-centered care: Postoperative	Structured phone or in-person interviews with patients and anesthesiologists to assess care after a cesarean section and to solicit feedback on a prototype mobile app for postoperative cesarean section care	App focusing on patient education and self-monitoring. 80% of patients interacted with the app >2x. Most accessed resources were controlling pain, an overview about days after surgery, and key contacts.
Soh et al 2019 [18]	RCT	42	Gastric cancer surgical patients	Patient-centered care: Postoperative	Alarm on the app every 60 min for 2 days with nurse dashboard versus app without alarms or dashboard. Primary outcome: ISI^v^ to assess the frequency of ISI use.	ISI was higher in the test group versus control but not significant (113.5 vs 93.2; *P=*.22). Active coughing showed significantly higher performance in the test group (107.8) compared with the control (94.8).
Sun et al 2014 [19]	Prospective	62	Pediatric patients (4-18 years)	Patient-centered care: Postoperative	Assessed pain with both paper FPS-R^w^ and CAS^x^ assessments as well as app-based (Panda) versions postsurgery. Primary outcome: correlation between scores.	Panda FPS-R scores correlated strongly with the original tool (r > .93). Panda CAS scores correlated strongly with the original CAS scores at both time points (r>0.87); mean pain scores were higher (up to plus 0.47) with Panda than with the original.
Warren-Stomburg et al 2016 [20]	Mixed	83	Adult surgical patients	Patient-centered care: Postoperative	Part I: paper-based questionnaire to identify patients’ attitudes towards follow-up techniques. Part II: feasibility test of a mobile app for follow-up.	42.2% prefer to respond by the mobile app in part I, but in part II, adherence to answering questions in the app was only 27.2%. Patients > 60 yrs prefer paper follow-up while patients <40 prefer telemedicine (*P=*.001).
Baumann et al 2019 [21]	Randomized crossover	74	Resident and attending anesthesiologists	Systems-based: Medication safety	Compared simulations with and without the app. Primary outcome: the probability of administering the accurate dosage.	The probability of “accurate” rated dosage was 77.7 (70.9-84.5%) in control versus 94 (90-97.8%) with the app.
Gorges et al 2019 [22]	Feasibility study	N/A	N/A	Systems-based	telePORT app designed with input from anesthesiology assistants for design features and work-domain analysis. Primary outcome: Usage patterns quantified.	telePORT is used more for help requests (approximately 4.5 per day) than team messaging (approximately 1 per day). OR monitoring was frequently utilized (34%). Loss of wireless connectivity was a barrier.
Haffey et al 2013 [23]	Retrospective	23	Opioid conversion apps	Systems-based: Medication safety	Apps with opioid dose conversion abilities identified. Dose calculations of seven commonly used opioid switches were compared between apps.	Of 23 apps, 52% had no stated medical professional involvement. There is a significant difference in mean conversion output for hydromorphone between apps with and without medical professional involvement (0.2256 vs 0.2536; *P=*.038).
Jabaley et al 2018 [24]	Cross-sectional	13,846	Anesthesiologists	Systems-based: Medication safety	A free mobile app (Anesthesiologist) deployed a 10-question survey about sugammadex use and related adverse events.	About 50% of anesthesia providers had access to sugammadex and were given a survey. Anaphylaxis rates are estimated to be between 0.005%-0.098%. 22.7% reported adverse drug reactions.
Lane et al 2012 [25]	Feasibility	40	Anesthesiologists	Systems-based	Development of VigiVU, a mobile app for OR awareness and communication, at Vanderbilt University Medical Center followed by beta testing done with a group of 40.	VigiVU push notifications to iPhone were faster than pager (mean 18 sec, SD 8.2). All beta users continued to use the app for the benefits of situational awareness in up to 4 ORs.
Lelaidier et al 2017 [26]	RCT	52	Anesthesia residents	Systems-based: Guideline compliance	Two simulated crises, with and without MAX app. Primary outcome: technical performance during crisis defined by the ESC^y^. Secondary outcome: nontechnical performance	Mean technical performance was higher in the MAX group vs. control group: 81.6%, SD 11.9) versus 58.6%, SD 10.8; *P<*.001 and nontechnical 33.7%, SD 4.4 versus 30.9%, SD 4.9 points; *P<*.001.
McEvoy et al 2016 [27]	RCT	259	Anesthesiologists	Systems-based: Guideline compliance	A 20-question test regarding clinical scenarios related to ASRA^z^ guidelines using ASRA Coag app versus any resource except the app (control). Primary outcome: test score	App group (92.4%, SD 6.6) scored higher than control (68.0%, SD 15.8; *P<*.001). App use increased the odds of selecting correct answers (7.8, 95% CI 5.7-10.7).
Rothman et al 2013 [28]	Cross-sectional	N/A	N/A	Systems-based	Message sent with VigiVu versus paging system. Primary outcome: transmission and receipt times calculated as their differences.	Mean latencies <1 sec for iPad and iPod devices and <4 sec for iPhone. Service performed better than third party paging systems (Aquis paging system had 0.6% incidence of prolonged message delivery, >100 sec)
Rubin et al 2017 [29]	Retrospective	N/A	Clinical anesthesia personnel	Systems-based: Medication Safety	Two years before intervention used as the baseline rates of adverse event reporting. Primary outcome: monthly reporting and same-day adverse event reporting compared before and after app implementation.	Median reported 12 events for the first year, 14 for the second year, and 20 after the introduction of the mobile app (*P*=.01). The rate of same-day reporting increased by 10% after the introduction of the app (*P*=.048).
Burstein et al 2018 [30]	Feasibility	N/A	N/A	Medical education	An app that to teaches Bier block.	It was implemented in 2015 with data collection until 2021.
De Oliveira et al 2013 [31]	RCT	20	Medical students	Medical education	Students were randomized to intervention (iLarynx app for 30 min) versus control (no iLarynx access). Primary outcome: the time required to advance fiberscope from the mouth up to the carina on a mannequin. Failed if carina not seen in <120 sec.	80% of the control group failed versus 20% in the intervention group (*P=*.01). 24 failed attempts in the control group and 4 in the iLarynx group (*P<*.005).
Linganna et al 2020 [32]	RCT	18	Anesthesiology residents	Medical education	Traditional intraoperative teaching of transesophageal echocardiography (control) vs access to EchoEducator app. Primary outcome: score increase from preintervention to postintervention assessment.	Intervention group demonstrated a greater increase in score; (plus 19.19%, 95% CI 4.14%-34.24%; *P*=.02) compared to control.

^a^RCT: randomized controlled trial.

^b^m-YPAS: modified Yale Perioperative Anxiety Scale.

^c^OR: operating room.

^d^PPV: pulse pressure variation.

^e^CO: cardiac output.

^f^dP/dt: max slope of pressure curve.

^g^NMB: neuromuscular block.

^h^TOF: train of four.

^i^N/A: not applicable.

^j^PPV_app_: pulse pressure variation by app.

^k^PPV_man_: pulse pressure variation by manual calculation.

^l^PPV_CAP_: pulse pressure variation using Capstesia.

^m^SVV_PC_: stroke volume variation using pulse contour analysis.

^n^PPV_PC_: pulse pressure variation using pulse contour analysis.

^o^CO_CAP_: cardiac output using Capstesia.

^p^CO_TD_: cardiac output using thermal dilution.

^q^AUROC: area under the receive operating characteristic

^r^VAS-100: visual analog scale-100.

^s^NRS-11: numeric rating scale-11.

^t^SwQoR: Swedish Quality of Recovery.

^u^RAPP: recovery assessment by phone points.

^v^ISI: incentive spirometer index.

^w^FPS-R: faces pain scale-revised.

^x^CAS: color analog scale.

^y^ESC: European Society for Cardiology.

^z^ASRA: American Society of Regional Anesthesia and Pain Medicine.

## Discussion

### Patient-Centered Care

Within anesthesia, the perioperative surgical home has arisen as a model that encompasses the goals of patient-centered care [33]. Mobile apps will play a large role in this area as they can directly engage patients before they arrive at the hospital and after their procedures. By providing on-the-go education, monitoring, and behavioral interventions, the anesthesia team can reduce patient anxiety preoperatively, increase communication through real-time mobile interactions perioperatively, and improve compliance with perioperative instructions.

#### Preoperative Interventions

Mobile apps provide an opportunity to decrease anxiety without additional sedatives in the pediatric population. Preoperative anxiety in pediatric populations is estimated to be as high as 50% [34]. Lee et al randomized 120 children between ages 1 and 10 (ASA I, II) who were undergoing elective surgery to receive intravenous midazolam (M), behavioral intervention through smartphone applications (eg, Soundtouch interactive, Pororo Sticker Book, Angry Bird) tailored to the child’s developmental status and preferences (S), or both (SM). While they found that anxiety using the modified Yale Preoperative Anxiety Scale (m-YPAS) was lower post-intervention for all three groups (M group: mean 52.8, SD 11.8 vs mean 41.0, SD 7.0; S group: mean 59.2, SD 17.6 vs mean 36.4, SD 7.3; SM group: mean 58.3, SD 17.5 vs mean 26.0, SD 3.4), the S group had lower anxiety levels relative to the M group (*P<*.01), and the SM group had the lowest level of anxiety (*P<*.01) [5].

In another randomized controlled trial (RCT) of 84 pediatric patients ages 4 to 8, the children’s median anxiety levels were lower for patients in the smartphone group (parents verbally informed of the anesthetic procedure and child received smartphone app) versus the control group (parent verbally informed about the anesthetic procedure only). The smartphone group had significantly lower m-YPAS scores compared to the control group, measured in the operating room (OR) before anesthesia induction (55.0 vs 23.4; *P<*.001) [4]. The ability to tailor mobile app choices to a child’s developmental stage demonstrates how mobile technology can be customizable for individual patients.

Another novel use for smartphone apps in the preoperative area is to improve dynamic communications using a centralized platform such as Listeo+. The Listeo+ app provides personalized information to patients, reminders for preoperative recommendations, and a channel for various provider communications. Listeo+ is currently being tested in a multicenter RCT to evaluate the percentage of compliance to preoperative recommendations, thereby decreasing the rate of surgery cancellations [35].

Mobile apps, such as a preoperative functional capacity app, can assist with preoperative risk assessment, even before the patient enters the health care facility. At the University of Chicago, the Step Test app integrates the Duke Activity Status Index (DASI) with the 6-minute walk test (6MWT) [6]. Step Test uses voice prompts and Apple’s CMPedometer to derive calculated step counts for the 6MWT. Rubin et al demonstrated that Step Test’s estimated steps exhibited good agreement with a research-grade pedometer in a cohort of 78 patients (intraclass correlation coefficient of 0.87; *P<.*001). The app facilitates efficient administration of DASI and 6MWT and provides immediate data to providers without occupying clinic staff. Future applications may include improving functional capacity through patient optimization before procedures. In summary, emerging mHealth apps demonstrate their potential to transform preoperative care by decreasing anxiety and providing coordinated, efficient care. Hopefully, in the future, providing an interface to optimize patient health status before surgery to improve outcomes.

#### Intraoperative Monitoring

Digital mHealth apps have also been tested as tools to enhance intraoperative monitoring. An ideal mobile app for intraoperative monitoring should be easy-to-use, accurate, noninvasive, cost-effective, and reliable. Capstesia (version 1.1.6; Galenic App SL) is an inexpensive and attractive smartphone app used to calculate pulse pressure variations (PPV) and cardiac output (CO) from a picture taken of the arterial waveform on a monitor screen. In a simulated environment comparing 408 pairs of PPV readings, Capstesia’s PPV demonstrated acceptable accuracy compared with the manual PPV if at least 3 photos of the waveform were taken (measurement error <12%). Accuracy was improved if it was averaged across 5 photos [9] but is less practical in the OR setting. Additional validation studies showed that Capstesia had a percentage of error of 20% for PPV and 13.8% for CO among 20 patients in the intensive care unit [7]. However, when the app was studied in 57 patients undergoing elective cardiac surgeries, its calculated PPV only weakly predicted fluid responsiveness (sensitivity of 73%, 95% CI 0.54-0.92, and a specificity of 74%, 95% CI 0.48-0.90) [11]. When comparing Capstesia’s PPV to stroke volume variation from an uncalibrated pulse wave analysis monitor, Joosten et al found that there was 79% overall agreement between the two, with a kappa coefficient of 0.55 [10]. These results demonstrate the promise and difficulties of creating a mobile app for real-world clinical intraoperative settings.

Digital mHealth apps have also shown promise in intraoperative neuromuscular blockade (NMB) assessment. Often, recovery of NMB is done subjectively due to the lack of accelerators [36,37] and can result in residual weakness and respiratory complications [38]. In a sample of 22 patients, Carvalho et al demonstrated a strong correlation between train-of-four ratios obtained by a standard accelerometer against an Android app used with the phone attached to the patient’s hand (R=0.98). The app also had a small mean difference (0.0004, 95% limits of agreement +/ 0.12) against standard accelerometry [8]. Thus, the digital app is a feasible way to assess NMB. However, further studies are needed to demonstrate efficacy before the widespread application of mobile apps as monitoring devices in the OR.

#### Postoperative Monitoring

There is an increasing focus on patient-reported outcome measures (PROM), alongside traditional biomarkers, in evaluating clinical care [39]. Mobile apps have emerged as an exciting new tool to track PROMs such as symptoms, pain, and satisfaction with health care delivery beyond the inpatient stay. Apps have been tested in clinical trials demonstrating initial feasibility and cost-effectiveness compared to the standard of care [13,15,16].

**Figure 1:**
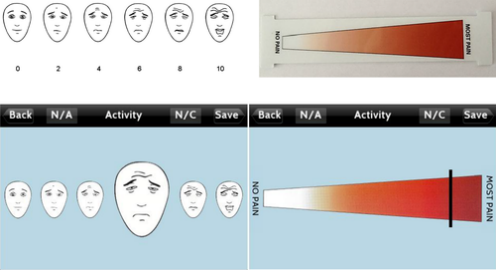
Original faces pain scale-revised (top) and adapted electronic faces pain scale-revised for Panda app (bottom) (reproduced with permission from John Wiley and Sons) [19].

One important measure that mobile apps help track is poorly managed acute postoperative pain, which can be related to increased risk of psychological morbidity, decreased quality of life (QoL), and chronic postsurgical pain [40]. The application Pain Assessment using a Novel Digital Application (Panda) contains electronic versions of the faces pain scale revised (FPS-R) and color analog scale (CAS). Figure 1 demonstrates both the original FPS-R and the electronic version seen on the app. In a prospective study of 62 patients, the app version correlated strongly with original scores at various postoperative time points (FPS-R: r>0.93; CAS: r>0.87) [19] and was preferable to the paper version (81% of FPS-R and 76% of CAS participants). Therefore, digital apps may improve adherence to self-reported pain. Additionally, in a study of 156 adult patients undergoing procedures commonly associated with postsurgical pain, Chiu et al found that the app version of the numeric rating scale 11 pain scores were equivalent to that of the paper version when used in adult patients following emergence from anesthesia and at discharge from the postanesthesia care unit [12]. The use of Panda in both adults and pediatric patients demonstrates the app's potential to be used in a variety of patient populations.

Another postoperative mobile app was built for patient-initiated inquiry to identify complications earlier and improve patients’ QoL after surgery. An RCT of 1027 day-surgery patients examined postoperative follow-up with a smartphone app known as recovery assessment by phone points (RAPP). The authors found that there were lower health care costs associated with smartphone app follow-up compared to standard of care follow-up (€60.69 vs €37.29; *P=*.008 with a mean difference of €23.66). However, after the intervention costs were included, net savings were only €4.77 per patient [13]. Patients using the RAPP also reported better global Swedish Quality of Recovery compared to patients using paper assessments on postoperative day 7 (28.23, SD 29.97 vs 34.87, SD 30.68; *P<*.001) and postoperative day 14 (20.12, SD 26.19 vs 21.90, SD 22.40; *P=*.002) [16]. The study also helped determine that most patient inquiries (62%) occurred 1 to 7 days postsurgery, most commonly related to the surgical wound (43/119, 36%) and pain (33/119, 28%) [14].

mHealth apps can also incentivize patients to improve respiratory mechanics postoperatively. The Go-breath app was tested in 42 patients who received general anesthesia undergoing a robotic or laparoscopic surgery or laparotomy to encourage the improved use of incentive spirometry (IS). Although it is a nice concept, patients who used the Go-breath app did not score statistically higher on the incentive spirometer index (frequency of IS use over two days) compared to patients who did not (113.5 vs 93.2; *P=*.22) [18].

Mobile apps can also provide a platform for multidisciplinary care in the postcesarean section period. For example, in a feasibility study including 15 patients of an app designed with obstetric anesthesiologists, 80% of patients used the app at least twice to self-monitor for complications and access pain control resources, thus increasing the amount of patient monitoring for common postcesarean complications and pain [17].

### Systems-Based Improvement

For anesthesiologists, smartphone apps are a convenient and accessible way to improve patient safety and quality of care [41,42] through medication safety, adverse event rates, guideline adherence, and responsiveness to emergencies.

For example, immediate response to OR emergencies is of utmost importance, with patient safety and outcomes on the line. Anesthesiologists at Vanderbilt have built an extensive iOS platform known as VigiVu to improve responsiveness through real-time OR videos, vital signs, anesthetic interventions, voice and text communication, and electronic medical record access [25]. Rothman et al demonstrated the superior reliability of the app’s push notifications compared to traditional paging systems—only 0.03% of iPhone notification latencies were over 100 seconds compared to 0.6% for third-party paging latencies [28]. Similarly, anesthesiologists at the University of British Columbia developed an iPhone app, telePORT, to support team-based communication and real-time OR monitoring. Initial results showed that telePORT was successfully integrated for OR help requests (4.5 requests per day) and OR monitoring, representing 34% of app visits [22]. The use of smartphone apps in these situations increases patient safety by prioritizing emergency situations and proper resource allocation. Additionally, data collection from patient monitoring and outcomes in mobile apps can also be utilized for both quality improvement and clinical research in the future.

#### Medication Safety and Adverse Event Reporting

Mobile apps have also been used to increase adverse event reporting through transparent and convenient processes that help further a culture of safety and quality. An iOS and Android adverse event–reporting app was developed at the University of Chicago (see figure 1 in Rubin et al [29] for an example of the iOS interface used to report events). After implementation, median monthly reporting rates for all providers increased from 12 to 20 (*P*<.001), with same-day reporting increasing by 10% during the intervention period (*P=*.048) [29]. Similarly, apps have shown efficacy in monitoring global adverse events regarding the administration of newer drugs. Jabaley et al repurposed a calculator app (Anesthesiologist) to distribute a survey regarding adverse drug reactions associated with sugammadex administration. Using the mobile app, the investigators gathered data from 2770 anesthesia providers that had experience administering the drug across 119 countries [24], demonstrating that an app may be used for crowdsourcing and surveilling new drugs. The app survey found that anaphylaxis rates were estimated to be between 0.005%-0.098%, and 22.7% of survey responders reported witnessing any sugammadex-related adverse drug reactions such as bradycardia and incomplete reversal of NMB [24].

Finally, anesthesiologists have historically used smartphones to calculate drug dosages. Baumann et al compared the probability of administering accurate medication dosages in emergency simulations with a dosage calculator app versus without it [21]. The probability of an accurate dose administration was higher in the app group compared to the control group (94%, SD 90-97.8 vs 77.7%, SD 70.9-84.5) [21]. Apps are often used for medication conversion in the outpatient pain management setting as well. For example, there are many opioid conversion applications designed to reduce medication errors. However, these apps should be used cautiously. In a study examining 23 opioid apps, only 50% provided direct references to sources for their conversion ratios, and over 50% had no documented medical professional involvement [23]. The conversion from 1 mg of oral morphine to oral hydromorphone was statistically different between apps with medical professional involvement versus those without (0.24 vs. 0.25; *P=*.04), demonstrating that there may be varying degrees of reliability for these mobile apps.

#### Guideline Compliance

Built-in smartphone-based decision support tools can similarly improve adherence to anesthesia guidelines to reduce technical errors. Numerous reports have found that poor application of guideline standards results in worse patient safety outcomes [43-45]. However, guidelines are numerous, lengthy, and cumbersome to follow. In an RCT across eight institutions, 259 anesthesiologists completed a test requiring the application of the American Society of Regional Anesthesia and Pain Medicine (ASRA) guidelines to clinical situations. The intervention group used an electronic decision support tool, ASRA Coags, which was programmed with the latest guidelines and decision logic, and the control group used any other resource. The authors found that the intervention group had a significantly higher score on a clinical scenario knowledge test (92.4 vs 68.0; *P<*.001) compared to the control group [27], irrespective of training. Together, these studies show the potential for mobile apps to act as a platform for the rapid application of complex guidelines in clinical situations, potentially reducing adverse events, preventing errors, and improving the quality of care.

Mobile apps have also been developed to improve performance in high-stress situations in lieu of posters, flow charts, and checklists. For example, MAX, a handheld cognitive aid app, was used as an intervention in a RCT of 52 anesthesia residents. Results demonstrated that residents performed better technically, as rated by independent observers when using MAX in simulated crises compared to without MAX use (81.6%, SD 11.9 vs 58.6%, 10.8; *P<*.001). The app also improved leadership (*P=*.003), problem-solving (*P<*.001), and resource-using (*P=*.006) as assessed by the Ottawa Global Rating scale [26].

### Medical Education

The landscape of medical education is changing from the traditional model of classroom teaching to models such as the flipped classroom and asynchronous learning. Mobile technology enables asynchronous learning—a time and location-independent learning model—with greater access to online modules, podcasts, and videos. For example, smartphone apps have now been developed for point-of-care learning of regional anesthetic procedures such as the Bier Block [30]. These apps may provide an all-in-one learning center with videos, cognitive aids, dose calculators, self-timers, and evidence-based references readily available at the learner's convenience [30]. Additionally, education apps use gamification or game-design elements to increase user motivation. Linganna et al developed EchoEducato*r*, a mobile app with transesophageal echocardiography image-based content, and tested it against traditional intraoperative teaching amongst 18 anesthesiology residents over two weeks (please see figure 2 in Linganna et al [32] for examples of questions testing pathology and structures as well as the feedback the resident receives). The app group had a greater knowledge increase than the control group (+19.19%, 95% CI 4.14-34.24; *P=*.02) in an assessment based on the perioperative transesophageal echocardiography exam [32]. Residents also reported that they would recommend the app to others because of its content customizability and convenience.

Another application of mobile apps in medical education is through simulation. For example, iLarynx utilizes the iPhone’s built-in accelerometer to mimic hand movements for fiberoptic intubation. When tested in 20 novice medical students, 80% of students in the standard training group had at least one failed attempt (>120 seconds) of visualizing the carina compared to 20% of students in the iLarynx group (*P=*.01). There was also continued group improvement in the iLarynx group but not in the standard training group [31].

### Current Obstacles and Future Directions

Despite the many functions that mobile technology could fulfill in anesthesia, various obstacles, including low patient participation, privacy and security, provider responsibility, and shifting workplace norms, ultimately impede their comprehensive integration into the clinical environment. For example, preoperative and postoperative monitoring interventions depend on patient interaction and motivation. While there are some reports of high adherence to mobile app interventions [15], other studies show a poorer response rate, especially among the elderly—a large hurdle in app development [20]. Intraoperative apps need to be assessed for ease of use, reliability, and accuracy in the OR and not distract anesthesiologists. Monitoring apps, although promising, need more clinical validation across a range of populations.

More evidence is needed to demonstrate mobile apps can meaningfully improve clinical outcomes moving forward. Large-scale, prospective studies with outcomes data are necessary to provide sufficient evidence for widespread implementation. Investigators should also consider using the mHealth evidence reporting and assessment guidelines, which address the complex nature of mobile technology research [46].

### Limitations

Similar to all narrative reviews, this review is limited by the lack of appraisal criteria for included studies. Additionally, only mobile apps derived from the scientific literature have been included. Thus, there are likely mobile applications apps not studied in the literature that were omitted. We have performed a qualitative review of the search and included only studies that fit into the categories we created, thus excluding other studies.

There are additional limitations to the mHealth apps described. Many are often inadequately tested clinically, lack efficacy data, and do not adhere to standard guidelines. Similarly, there are limited guidelines and protocols on how to use the data collected by these apps, despite their potential for engaging a wide population. Future research should explore if patient engagement through smartphone apps may increase efficiency, patient satisfaction, and outcomes during the perioperative period.

### Conclusion

With the rapid uptake of smartphones amongst patients and clinicians alike, mobile applications will likely play a larger role within anesthesiology in the future. mHealth apps have novel roles in improving patient care, efficiency, and intraoperative monitoring during surgery. Mobile apps have also been shown to facilitate systems-wide change by creating a culture of improving patient quality and safety. However, as the field of anesthesiology moves forward into the digital health space, demonstrating feasibility is not sufficient; clinicians must critically evaluate mobile app study protocols and rigor. Despite studies with smaller populations and simulated environments, this review still finds emerging evidence that mHealth applications have the potential to significantly improve communication between anesthesiologists, improve workflow efficiency, enhance medical education, and reduce hospitals’ costs in the perioperative arena.

## Abbreviations

6MWT: 6 Minute Walk Test
ASRA: American Society of Regional Anesthesia
CAS: color analog scale
CO: cardiac output
DASI: Duke Activity Status Index
FPS-R: faces pain scale-revised
IS: incentive spirometry
ICC: intraclass correlation coefficients
mHealth: mobile health
mYPAS: modified Yale Preoperative Anxiety Scale
NMB: neuromuscular blockade
OR: operating room
PPV: pulse pressure variation
PROM: patient-reported outcome measures
QoL: quality of life
RAPP: recovery assessment by phone points
RCT: randomized controlled trial
